# Higher Dietary Inflammatory Index Scores Are Associated With Stress and Anxiety in Dormitory-Residing Female University Students in the United Arab Emirates

**DOI:** 10.3389/fnut.2022.814409

**Published:** 2022-03-10

**Authors:** Amita Attlee, Coumaravelou Saravanan, Nitin Shivappa, Michael D. Wirth, Mashael Aljaberi, Reem Alkaabi, Mo'ath F. Bataineh, James R. Hebert

**Affiliations:** ^1^Department of Nutrition and Health, College of Medicine and Health Sciences, United Arab Emirates University, Al Ain, United Arab Emirates; ^2^Department of Clinical Psychology, School of Rehabilitation and Behavioral Sciences, Vinayaka Mission's Research Foundation (DU), Aarupadai Veedu Medical College and Hospital Campus, Pondicherry, India; ^3^Department of Epidemiology and Biostatistics, Cancer Prevention and Control Program, Arnold School of Public Health, University of South Carolina, Columbia, MO, United States; ^4^Connecting Health Innovations LLC, Columbia, MO, United States; ^5^College of Nursing, University of South Carolina, Columbia, MO, United States; ^6^Department of Sport Rehabilitation, College of Physical Education and Sport Sciences, The Hashemite University, Zarqa, Jordan

**Keywords:** anxiety, depression, Dietary Inflammatory Index, dormitory, stress, students, university

## Abstract

Dormitory-residing university students are at-risk of mental health problems related to unhealthy diets. The purpose of this study was to assess the association between dietary inflammatory potential and mental health of dormitory-residing female university students. This cross-sectional study was comprised of 260 undergraduate females residing in dormitories of the largest university in United Arab Emirates during Spring 2019. The Energy-adjusted Dietary Inflammatory Index (E-DII^TM^) scores calculated from 37 food parameters were derived from two 24-h dietary recalls. The logistic regression analysis was used to estimate odds ratios (ORs) and 95% CIs for the E-DII score in relation to depression, anxiety, and stress. The E-DII scores (mean = 2.98 ± 1.17) were categorized into: tertile 1 (−1.96–2.62), tertile 2 (2.63–3.52), and tertile 3 (3.53–5.60), representing less to more proinflammatory diets. Students in the E-DII tertile 3 had significantly higher depression, anxiety, and stress scores. The logistic regression analysis showed that each point increase in the E-DII score was associated with symptoms of stress (OR = 1.41; 95% CI: 1.12–1.77; *p* = 0.003) and anxiety (OR = 1.35; 95% CI: 1.07–1.69; *p* = 0.01). Relative to students in the E-DII tertile 1, those students in the E-DII tertile 3 were more likely to be at higher risk of stress and anxiety OR_E−DIItertile3vs1_ = 2.89 (1.44–5.79) and 2.88 (1.49–5.56), respectively. Overall, stress and anxiety were associated with proinflammatory diets in dormitory-residing female university students, suggesting the need for targeted interventions to increase the anti-inflammatory capacity of diet and improve mental wellbeing in students on university campuses.

## Introduction

University students are considered a relatively healthy segment of population, although they are at an increased risk of physical and mental health issues (1, 2). Erratic schedules and academic stress often lead to unhealthy lifestyles and food choices such as overconsumption of calorie-dense foods rich in fats and simple carbohydrates, lower consumption of nutrient-dense foods such as fruits and vegetables, and irregular eating schedules or entirely skipping meals (3). One-third of the university students in United States reported at least one metabolic abnormality associated with their health-related habits (1). Higher rates and frequencies of physical health problems and mental health disorders have been reported in female than male university students (4, 5). This is consistent with other research showing that female medical students in the United States tend to have higher rates of depression than male medical students (6). Poor dietary habits pose serious threats to an increased risks of obesity in university students (7). Chronic diseases such as type 2 diabetes mellitus (T2DM), cardiovascular disease (CVD), asthma, and chronic bronchitis are common (16.5–33.5%) among university students (8). Additionally, long study hours, sleep deprivation, and inadequate personal time may result in adverse consequences on mental wellbeing of university students (9). The WHO World Mental Health Surveys reported anxiety in 11.7–14.7% of university students (2). Relatively high prevalence of depression (62.2%), anxiety (78.3%), and stress (55.9%) was reported among university students in Malaysia (5). Awadalla et al. (10) found depression in 34.2% and anxiety in 22.3% of university students in United Arab Emirates (UAE) (10).

Diet and nutrition are uniquely involved in mental health abnormalities, due to the ability of specific food constituents to influence inflammatory pathways (11) that contribute to chronic diseases (12–14) and mental health disorders (15). There is growing interest in modulating inflammation to reduce cardiometabolic risks (16) and mental health outcomes (17) by altering intakes of pro- and anti-inflammatory food components of usual diets. Positive associations have been reported between body composition, chronic stress, low-grade chronic inflammation, and intakes of certain dietary components in Croatian university students (18).

The Dietary Inflammatory Index (DII®) was developed in 2009 (19) and updated in 2014 with the goal of providing a quantitative measure of dietary inflammatory potential and a predictor of various chronic health conditions (20). The DII is based on pro- and anti-inflammatory effects of specific dietary components, including macronutrients, vitamins, minerals, flavonoids, and specific foods. The DII scores are calculated based on a method that compares the individual's reported intakes to a global comparative database, thus allowing their use across different cultures and dietary patterns and produces scores directly comparable across all the human populations (21).

In addition to the positive association between the DII score and risk of cardiometabolic diseases (22), a comprehensive evaluation of current evidence indicated that higher DII was associated with an increased risk of common mental health outcomes, including depression, symptoms of anxiety, and distress, as well as schizophrenia in adults (23).

Ironically, there is limited literature on the association between dietary intakes and mental health of university students residing in dormitories who are likely to be more adversely affected than their counterparts residing off-campus with their families. These students may face greater stressful situations arising from their relationship with other residents and staff, time management and adjustment to the dormitory environment (24) that may have implications on their dietary intakes and mental health. Therefore, this study was designed to focus on dietary inflammatory potential in relation to mental health status among female students residing in university dormitories in UAE. As of the time this article was written, the authors were not able to identify similar studies conducted in other countries.

## Materials and Methods

### Participants and Study Design

The United Arab Emirates University (UAEU), founded in 1976, is the first comprehensive national university in the UAE. It offers programs through nine colleges, with two of them—College of Medicine and Health Sciences and College of Food and Agriculture—offering medical- and health-related majors. As per the university statistics of 2018–2019, a total of 15,091 students were registered in different undergraduate and graduate programs. The majority, 81%, of the students were females and nearly 40% of the registered undergraduate students resided in university dormitories, thus constituting a large proportion of on-campus residential population.

The UAEU has eight student dormitories that provide three meals on five working days (Saturday night through Thursday morning) per week to all the dormitory residents. However, the international students reside in two dormitories that operate regularly during weekends and semester breaks as well. At the UAEU, students come from different parts of the world with diverse cultural backgrounds. Different cuisines such as Arabic, Asian, Italian, and other Western cuisines are served by the UAEU Food Services (FSs). Only Halal (prepared as prescribed by Islamic law) foods are served. The FS operation follows a 3-month cycle menu; however, special menus are offered during the Islamic month of Ramadan and Islamic festivals (Eids). In addition to the regular menu items, FS offers “diet food” section, where students can choose from low-calorie and mildly flavored foods. It also caters to the students with special dietary restrictions and recommendations according to the medical advice for allergies or other medical conditions. The FS in dormitories follows fixed mealtime schedule and fixed portions (for cooking) with the flexibility of increasing or reducing (for service) according to the student's choice. Additionally, the students have access to a variety of commercial food outlets in the “food court” under the university's students' campus life unit.

For this study, a cross-sectional research design was adopted. Female students registered in undergraduate programs, residing in the UAEU dormitories, eating at least one main meal (breakfast/lunch/dinner) on most of the weekdays in dormitory (FS) canteen were selected. The minimum age for participation was 18 years and pregnancy/lactation in any of them during this study period was also considered. However, a student on medical treatment for a mental health condition (such as depression, anxiety, stress, and eating behavior disorder) or on a special diet for any chronic condition such as obesity, hypertension, or diabetes was excluded. Posters, emails, announcements, and word of mouth strategies were used to invite the students to participate in this study. Interested students were requested to carefully read the participant information sheet. Research investigators fixed prior appointments with them for carrying out assessments and administering 24-h recall questionnaire in the Department of Nutrition and Health laboratory, UAEU after obtaining written informed consents. Additionally, research investigators conducted telephonic follow-ups to complete the repeated 24-h recalls. Further, the participants self-administered paper version of physical activity and mental health questionnaires. Research investigators checked the completeness and correctness of responses and clarified any doubt before the completed questionnaires were returned. Data collection took place in Spring semester between March 2019 and May 2019.

Using G^*^Power software and based on power of 95 and a 5% type error rate (corresponding to a 95% CI) and accounting for 10 independent predictors, a minimum of 172 participants were required to detect moderate effect sizes of diet on mental health outcomes. For this study, 260 students were recruited using convenience sampling methodology, wherein any interested student meeting the inclusion criteria was selected to reach the target sample size. The UAEU Human Research Ethics Committee (ERH-2019-5870-19-04) granted ethical approval for this study.

### Survey Items

#### Participant Characteristics

The participant information included the student's age, nationality, study major (medical and health related and others), marital status, and whether they were pregnant or lactating.

#### Physical Health Attributes

Self-rated health and medical condition (any chronic medical illness diagnosed or actively receiving treatment for CVD, hypertension, diabetes, cancer, hormonal imbalance, psychological disorder, or any other in the past 3 months or more) were asked. Additionally, body habitus, smoking status, and physical activity level were measured as physical health attributes of the participants.

#### Body Habitus

Height, weight, and waist circumference (WC) were measured using standardized techniques (25). Height was measured to the nearest 0.10 cm using a stadiometer (Seca 213, capacity 205 cm, Seca Company, USA). Weight was measured to the nearest 100 g with participants in light clothing and without shoes using a digital weighing scale (Seca 872, capacity 150 kg, Seca Company, USA). WC was measured to the nearest 0.10 cm, using a measuring tape (Seca 201, capacity 205 cm, Seca Company, USA) at the midpoint between the lower costal margin and the iliac crest. The WHO classification categorized the body mass index (BMI) values as underweight (<18.50 kg/m^2^), normal (18.50–24.99 kg/m^2^), overweight (25.00–29.99 kg/m^2^), or obese (≥30.00 kg/m^2^) and cutoff of WC > 80 cm indicated central obesity (26).

#### Smoking Status

Participants were categorized as current, former, and never smokers based on the standard WHO criteria (27, 28).

#### Physical Activity

The students completed the self-administered bilingual (English and Arabic) 27-item International Physical Activity Questionnaire (IPAQ-27)—long version. The validity and reliability of the IPAQ-27 long version in English and in Arabic were previously established (29, 30). The activity duration was used to calculate metabolic equivalent of tasks (METs)/week categorizing physical activity levels into sedentary, light, moderate, or high, as previously suggested (29, 30).

#### Dietary Inflammatory Index

For determining the DII scores, usual dietary intakes of students were assessed using 2-day repeated 24-h dietary recall method. One-to-one interviews (direct and/or telephone) were conducted to ask the students about their intakes of all the food items and beverages in the last 24 h on 1 weekday (WD) and 1 weekend (WE) day, respectively (25). Detailed information on supplements and certain spices was also taken. The nutrient analysis was performed using Food Processor Diet Analysis and Fitness Software version 10.11 (ESHA Research Incorporation, Salem, USA). Some indigenous Arabic/Emirati dishes not included in the ESHA database were created in the database by formulating the recipe for composite dishes using either the available ingredients in the software or obtaining nutritional values from local food composition tables and entering them into the ESHA database.

The UAEU FS catering provides food for the students residing in university dormitories, which facilitated portion-size estimates and reduced errors in the nutrient composition analysis of composite dishes due to the known amounts of ingredients of the typical WD diets of the students. A repeated assessment on the WE day further provided a better estimate of their usual dietary intakes.

The development and validation of the DII scores were explained in detail elsewhere (19–21). To elaborate, the DII scores are based on forty-five food parameters that constitute nutrients, spices, and whole foods. Briefly, the dietary data of the sample were first linked to the world database that provided a robust estimate of a mean and SD for each parameter. This was achieved by subtracting the “standard global mean” from the reported intake and dividing this value by the SD to generate “z” scores. To minimize the effect of “right skewing,” these “z” scores were then converted to a centered proportion. The centered proportion for each food parameter for each individual was then multiplied by the respective food parameter article effect score (inflammatory potential for each food parameter), which was derived from the literature review. All the food parameter-specific DII scores were then summed to create the overall DII score for each participant in this study. Finally, the Energy-adjusted DII (E-DII) scores were calculated using the density method wherein all the food parameters were converted to nutrients consumed per 1,000 kcal and the same procedure was used to relate individual exposure data to the global energy-adjusted database (31, 32). In this study, thirty-seven food parameters were considered, excluding eight food parameters (flavones and isoflavone compounds) due to insufficient data in the food composition databases and alcohol due to cultural irrelevance. Proinflammatory components included: energy, carbohydrate, protein, total fat, saturated fat, iron, cholesterol, *trans* fat, and vitamin B12; while anti-inflammatory components included: fiber, monounsaturated fatty acids, polyunsaturated fatty acids, ω-3 fatty acids, ω-6 fatty acids, vitamin A, vitamin B1, vitamin B2, vitamin B3, vitamin B6, vitamin C, vitamin D, vitamin E, magnesium, zinc, selenium, folic acid, and caffeine; and specific food items (all anti-inflammatory): garlic, ginger, pepper, onion, rosemary, saffron, black/green tea, thyme, turmeric, and cloves (eugenol) were included.

Food and nutrient intakes of the students were calculated for WD and WE days separately. Average intake from the 2-days repeated diet recalls was considered as the usual intake for which the E-DII scores were then calculated. The higher E-DII scores indicate higher proinflammatory potential and lower scores reflect more anti-inflammatory effect of the diet (32).

#### Mental Health

The self-reported bilingual 21-item Depression, Anxiety, and Stress Scale (DASS-21) was employed to assess the mental state of students experienced in the past 1 week according to three subscales: depression, anxiety, and stress (33, 34). Accordingly, the items are scored on a 4-point scale ranging from 0 (did not apply to me at all) to 3 (applied to me very much or most of the time). Participants were classified into showing either presence or absence of mental health symptoms, with presence of symptoms defined as a depression score > 9 (range = 0–42; a higher score indicates an increased severity of symptoms), an anxiety score > 7 (range = 0–42; a higher score indicates an increased severity of symptoms), and/or a stress score > 14 (range = 0–42; a higher score indicates an increased severity of symptoms), as previously described. The Cronbach's alpha coefficients for the English version of the DASS-21 were 0.81, 0.89, and 0.78 for depression, anxiety, and stress subscales, respectively (33) and the Cronbach's alpha coefficients for the Arabic version of the DASS-21 were 0.81, 0.76, and 0.77 for depression, anxiety, and stress subscales, respectively (34).

### Statistical Analysis

Data analysis was performed using IBM SPSS® Statistics version 28 (SPSS Incorporation, Chicago, Illinois, USA). Normality of data was tested using the Kolmogorov–Smirnov test. The mean E-DII score of the students was 2.98 ± 1.17 (range −1.96– +5.60). The students were divided into the tertiles of the E-DII scores calculated based on the distribution of the E-DII scores as follows: tertile 1 (−1.96–2.62), tertile 2 (2.63–3.52), and tertile 3 (3.53–5.60). Tertiles 1, 2, and 3 indicated low, medium, and high proinflammatory diets, respectively.

Descriptive statistics were used for the participants' characteristics, body habitus, smoking status, physical activity, and mental health measures across the E-DII tertiles. Frequency distributions in terms of numbers and percentages were calculated for the categorical variables and the chi-squared test was used to estimate the associations of participants' characteristics and health attributes across the E-DII tertiles including BMI and WC categories, smoking and physical activity categories, and presence of self-reported symptoms of depression, anxiety, and stress. Means and SDs and medians and interquartile ranges (IQRs) were calculated for continuous variables. The non-parametric Kruskal–Wallis *H* one-way ANOVA was used for comparing the significance of differences in the BMI, WC, energy and nutrients intakes and food groups, and mental health measures (depression, stress, and anxiety) across the tertiles of the E-DII scores.

The logistic regression analysis was used to detect the associations of the E-DII with depression, anxiety, and stress. The univariate analysis was conducted to select the model and only independent variables with *p* < 0.20 were included in the final model. Body habitus measures (BMI and WC), nutrient intakes and specific food groups, smoking status, physical activity categories, and the E-DII were fit as the independent variables. Odds ratio (OR) and 95% CIs were calculated.

The logistic regression analysis was fit with dichotomously categorized depression, anxiety, or stress as the dependent (i.e., outcome) variable to determine the association with the E-DII score. The E-DII score, fit as an independent variable, was analyzed as continuous independent variable and categorized into tertiles. Tertile 1 (reference group) represented the most anti-inflammatory diet and tertile 3 represented the most proinflammatory diet. ORs were calculated after controlling for age, BMI, WC, whether the student was Emirati by nationality, presence of chronic medical condition, smoking status, and self-rated health. All the statistical tests were assigned to type I error rate of α = 0.05 as the nominal level of significance.

## Results

The internal reliability for the bilingual English and Arabic versions of the DASS-21 and the IPAQ-27 item scales used in this study was high (Cronbach's alpha coefficients > 0.70). Table 1 shows the distribution of participants' characteristics according to the tertiles of the E-DII scores. The average E-DII score in this population was 2.98 ± 1.17, with a range of −1.96–5.60. The mean age of study participants was 20.25 ± 1.77 years. Out of 260 female students, 88.1% were UAE Nationals (Emirati) and the remaining 11.9% expatriates were mostly from other Arab countries, with a few from South Asia. The students were registered in either medical- and health-related majors (30.0%) or others (70.0%) such as education, humanities, social sciences, law, engineering, and information technology. The majority (96.2%) of the students were single and none of the married students were pregnant or lactating during this study period. No significant associations were found in the students' age, nationality, study major, self-rated health, marital status, medical conditions, BMI, WC, and physical activity levels across the tertiles of the E-DII score. However, average scores of the DASS-21 subscales—depression, anxiety, and stress were above their cutoffs for normal, indicating poor mental health status among dormitory-residing female university students. The chi-square analysis revealed significant associations for anxiety (χ^2^ = 13.98, *p* = 0.001) and stress (χ^2^ = 13.34, *p* < 0.001) with the E-DII tertiles.

**Table 1:** Participants' characteristics and physical and mental health measures across the tertiles of the Energy-adjusted Dietary Inflammatory Index (E-DII) scores (*n* = 260).

**Variable**	**E-DII Score**				***p*-value**
	**Total**	**Tertile 1 (−1.96–2.62)**	**Tertile 2 (2.63–3.52)**	**Tertile 3 (3.53–5.6)**	
	**(*n* = 260)**	**(*n* = 88)**	**(*n* = 88)**	**(*n* = 84)**	
Age (years), Mean ± SD	20.3 ± 1.8	20.1 ± 1.5	20.3 ± 2.0	20.5 ± 1.8	0.36
Age (years), Median (IQR)	20 (19–21)	20 (19–21)	20 (19–21)	20 (19–22)	0.36
**Nationality**, ***n*** **(%)**					
Emirati	229 (88.1)	77 (87.5)	75 (85.2)	77 (91.7)	0.42
Other	31 (11.9)	11 (12.5)	13 (14.8)	7 (8.3)	
**Study major**, ***n*** **(%)**					
Medicine/Health-related	78 (30.0)	25 (28.4)	28 (31.8)	25 (29.8)	0.88
Other	182 (70.0)	63 (71.6)	60 (68.2)	59 (70.2)	
**Marital status**, ***n*** **(%)**					
Single	250 (96.2)	86 (97.7)	84 (95.5)	80 (95.2)	0.64
Married	10 (3.8)	2 (2.3)	4 (4.5)	4 (4.8)	
Body mass index (kg/m^2^), mean ± SD	23.0 ± 5.6	23.1 ± 5.7	23.2 ± 6.0	22.7 ± 4.8	0.99
Body mass index (kg/m^2^), median (IQR)	21.3 (19.7–25)	21.3 (19.5–25.6)	21.3 (19.9–24)	21.3 (19.6–25.2)	0.99
**Body mass index categories**, ***n*** **(%)**					
Underweight	35 (13.5)	13 (14.8)	12 (13.6)	10 (11.9)	0.92
Normal	180 (69.2)	58 (65.9)	61 (69.3)	61 (72.6)	
Overweight/Obese	45 (17.3)	17 (19.3)	15 (17.0)	13 (15.5)	
Waist circumference (cm), mean ± SD	74.8 ± 12.6	75.1 ± 13.4	75.0 ± 13.3	74.3 ± 10.9	0.96
Waist circumference (cm), median (IQR)	74.3 (65.4–82.2)	74.6 (65.5–81.1)	72.9 (65–84.8)	75.3 (66–82.8)	0.96
**Waist circumference categories**, ***n*** **(%)**					
Normal	186 (71.5)	64 (72.7)	62 (70.5)	60 (71.4)	0.95
Central Obese	74 (28.5)	24 (27.3)	26 (29.5)	24 (28.6)	
**Self-rated health**, ***n*** **(%)**					
Poor/fair	113 (43.5)	42 (47.7)	30 (34.1)	41 (48.8)	0.27
Good	79 (30.4)	24 (27.3)	30 (34.1)	25 (29.8)	
Very good/excellent	68 (26.2)	22 (25.0)	28 (31.8)	18 (21.4)	
**Medical condition**, ***n*** **(%)**					
Yes	30 (11.5)	8 (9.1)	10 (11.4)	12 (14.3)	0.57
No	230 (88.5)	80 (90.9)	78 (88.6)	72 (85.7)	
**Smoking status**					
Yes	36 (13.8)	6 (6.8)	13 (14.8)	17 (20.2)	0.04
No	224 (86.2)	82 (93.2)	75 (85.2)	67 (79.8)	
**Physical activity**, ***n*** **(%)**					
Sedentary	55 (21.2)	12 (13.6)	24 (27.3)	19 (22.6)	0.42
Low	133 (51.2)	47 (53.4)	43 (48.9)	43 (51.2)	
Moderate	43 (16.5)	16 (18.2)	13 (14.8)	14 (16.7)	
High	29 (11.2)	13 (14.8)	8 (9.1)	8 (9.5)	
Depression score, mean ± SD	11.3 ± 8.4	9.3 ± 7.2	11.3 ± 8.4	13.4 ± 9.1^§^	0.008
Depression score, median (IQR)	10 (4–16)	8 (4–14)	10 (4–16)	12 (6–18) ^§^	0.008
**Depression symptoms**, ***n*** (%)^*^					
Yes	135 (51.9)	40 (45.5)	47 (53.4)	48 (57.1)	0.29
No	125 (48.1)	48 (54.5)	41 (46.6)	36 (42.9)	
Anxiety score, mean ± SD	10.8 ± 7.9	8.9 ± 6.5	9.8 ± 7.8	13.7 ± 8.5 ^§^^,^^**^	<0.001
Anxiety score, median (IQR)	10 (4.5–14)	8 (4–12)	8 (4–14)	12 (8–20) ^§^^,^^**^	<0.001
**Anxiety symptoms**, ***n*** (%)*					
Yes	162 (62.3)	48 (54.5)	48 (54.5)	66 (78.6)	0.001
No	98 (37.7)	40 (45.5)	40 (45.5)	18 (21.4)	
Stress score, mean ± SD	14.5 ± 8.9	11.3 ± 7.1	14.2 ± 8.5	18.1 ± 9.6 ^§^^,^^**^	<0.001
Stress score, median (IQR)	14 (8–18)	10 (6–16)	14 (8–18)	16 (10–25.5)^§^^,^^**^	<0.001
**Stress symptoms**, ***n*** (%)*					
Yes	155 (59.6)	40 (45.5)	54 (61.4)	61 (72.6)	0.001
No	105 (40.4)	48 (54.5)	34 (38.6)	23 (27.4)	

*SD, Standard Deviation; IQR, Interquartile Range*.

* *Depression>9, anxiety>7, stress >14*.

§ *Tertile 3 is significantly different than tertile 1, p < 0.05*.

** *Tertile 3 is significantly different than tertile 2, p < 0.05*.

The results of Kruskal–Wallis *H* analysis for the DASS-21 subscales (depression, anxiety, and stress) as dependent variables and the E-DII tertiles as the independent variable also revealed significantly higher scores for all the DASS-21 subscales in tertile 3 as compared to participants in tertile 1 and/or tertile 2 (*p* < 0.05). All the DASS-21 subscales scores were comparable between tertile 1 and tertile 2.

Table 2 shows the results of Kruskal–Wallis *H* analysis for nutrient and dietary intakes of students as dependent variables across the tertiles of the E-DII scores as independent variable. As expected, the intakes of the following proinflammatory nutrients: energy, total fat, saturated fatty acids, and cholesterol as well as grains food group were significantly higher among students in tertile 3 of the E-DII scores. In contrast, intakes of anti-inflammatory nutrients including mono- and polyunsaturated fatty acids and vitamin E were significantly higher among students in tertile 2 of the E-DII scores.

**Table 2:** Comparison (mean ± SD) of daily nutrient and dietary intakes across the tertiles of the E-DII scores (*n* = 260).

**Variable**	**E-DII score**				
	**Total**	**Tertile 1**	**Tertile 2**	**Tertile 3**	***p*-value**
	**(*n* = 260)**	**(*n* = 88)**	**(*n* = 88)**	**(*n* = 84)**	
Energy (Kcal)	2,054.5 ± 549.9	1,928.9 ± 556.9	2,055.9 ± 529.8	2,184.7 ± 539.1^§^	0.009
Protein (g)	68.1 ± 24.8	67.2 ± 24.3	70.0 ± 23.0	67.0 ± 27.1	0.67
Carbohydrate (g)	244.9 ± 85.8	243.6 ± 85.4	247.8 ± 81.4	243.3 ± 91.6	0.93
Fiber (g)	15.6 ± 7.5	15.3 ± 6.7	16.6 ± 7.8	15.0 ± 7.8	0.29
Total Fat (g)	52.5 ± 21.1	46.0 ± 18.5	52.2 ± 18.1	59.8 ± 24.4^§^^,^^**^	<0.001
Saturated Fatty acid (g)	29.3 ± 13.9	24.7 ± 9.2	25.3 ± 7.9	38.2 ± 18.2^§^^,^^**^	<0.001
Monounsaturated Fatty acid (g)	15.3 ± 9.1	14.4 ± 8.8	17.3 ± 8.7^¥^	14.3 ± 9.7	0.048
Polyunsaturated Fatty acid (g)	6.7 ± 6.7	5.7 ± 5.1	8.5 ± 9.0 ^¥^	5.8 ± 4.9^**^	0.007
Trans Fat (g)	1.3 ± 1.3	1.1 ± 1.2	1.1 ± 1.1	1.5 ± 1.6	0.09
Cholesterol (mg)	212.7 ± 121.0	174.7 ± 87.9	223.6 ± 119.5^¥^	241.2 ± 141.9^§^	0.001
Omega 6 Fatty Acid (g)	4.0 ± 3.2	3.8 ± 3.0	4.4 ± 3.5	3.8 ± 3.2	0.33
Omega 3 Fatty Acid (g)	0.5 ± 0.4	0.5 ± 0.4	0.5 ± 0.4	0.4 ± 0.3	0.23
Vitamin A (IU)	3,921.6 ± 3,955.7	3,606.2 ± 3,044.3	3,696.2 ± 3,327.1	4,488.1 ± 5,198.8	0.28
Vitamin B_1_ (mg)	1.1 ± 1.0	0.9 ± 0.9	1.2 ± 1.1	1.1 ± 1.0	0.14
Vitamin B_2_ (mg)	1.4 ± 5.3	2.2 ± 9.0	1.0 ± 0.6	0.9 ± 0.7	0.18
Vitamin B_3_ (mg)	11.6 ± 8.3	10.6 ± 6.8	13.1 ± 9.5	11.2 ± 8.2	0.12
Vitamin B_6_ (mg)	0.6 ± 0.6	0.6 ± 0.5	0.7 ± 0.6	0.6 ± 0.6	0.48
Vitamin B_12_ (μg)	1.6 ± 3.8	1.0 ± 0.9	1.8 ± 4.7	2.2 ± 4.6	0.096
Vitamin C (mg)	74.4 ± 95.8	74.0 ± 108.4	76.7 ± 92.2	72.4 ± 85.9	0.49
Vitamin D (IU)	44.0 ± 43.8	43.0 ± 43.2	47.8 ± 44.3	41.0 ± 43.9	0.58
Vitamin E (IU)	4.7 ± 4.6	4.5 ± 4.8	5.7 ± 5.6^¥^	4.0 ± 2.6	0.04
Folate (μg)	194.3 ± 138.7	180.6 ± 133.9	205.3 ± 127.5	197.1 ± 154.6	0.49
Calcium (mg)	804.5 ± 339.6	800.4 ± 353.9	838.3 ± 321.8	773.3 ± 343.4	0.45
Iron (mg)	11.7 ± 6.9	10.6 ± 6.8	12.5 ± 6.8	12.1 ± 6.9	0.15
Magnesium (mg)	111.8 ± 69.4	104.2 ± 61.6	121.3 ± 77.1	109.9 ± 68.3	0.25
Zinc (mg)	3.5 ± 3.3	3.2 ± 2.5	4.0 ± 3.2	3.4 ± 4.1	0.23
Grains (Serving)	6.1 ± 3.0	5.6 ± 2.9	5.8 ± 2.6	7.1 ± 3.2^§^	0.001
Vegetables (Serving)	1.6 ± 1.2	1.6 ± 1.4	1.6 ± 1.2	1.4 ± 1.1	0.29
Fruits (Serving)	0.7 ± 0.9	0.6 ± 0.8	0.6 ± 0.8	0.8 ± 1.0	0.28
Dairy (Serving)	1.6 ± 1.0	1.6 ± 1.0	1.7 ± 1.0	1.5 ± 0.9	0.67
Protein (Serving)	4.5 ± 2.6	4.4 ± 2.4	4.6 ± 2.6	4.6 ± 2.7	0.75

*SD, Standard Deviation; IQR, Interquartile Range*.

¥ *Tertile 2 is significantly different than tertile 1, p < 0.05*.

§ *Tertile 3 is significantly different than tertile 1, p < 0.05*.

** *Tertile 3 is significantly different than tertile 2, p < 0.05*.

Table 3 shows the associations between tertiles of the E-DII with the presence of symptoms for depression, anxiety, and stress. The logistic regression analysis revealed a significant positive association between the E-DII as a continuous variable and both the stress and anxiety. The results were further confirmed using tertiles of the E-DII, as participants in the E-DII tertile 3 were more likely to have symptoms of stress and anxiety than those in the E-DII tertile 1. However, the analysis revealed that those participants in the E-DII tertile 2 were not at a statistically significantly higher risk of stress and anxiety in comparison with tertile 1. Also, no association between the E-DII score and depression was evident.

**Table 3:** Adjusted odds ratios (ORs) and 95% CIs obtained from the logistic regression analysis for the E-DII association with the presence of depression, anxiety, and/or stress symptoms.

	**Tertiles of E-DII**						**E-DII (continuous)**		
	**Tertile 2 (Tertile 1** **=** **reference group)**			**Tertile 3 (Tertile 1** **=** **reference group)**			**OR**	**CI**	***p***
	**OR**	**CI**	***p***	**OR**	**CI**	***p***			
Depression (>9)	1.36	0.72–2.56	0.34	1.47	0.78–2.77	0.23	1.04	0.84–1.30	0.72
Anxiety (>7)	0.97	0.52–1.82	0.93	2.89	1.44–5.79	0.003	1.35	1.07–1.69	0.01
Stress (>14)	1.80	0.96–3.39	0.07	2.88	1.49–5.56	0.002	1.41	1.12–1.77	0.003

*Controlling for age, BMI, WC, whether the student was Emirati by nationality, presence of chronic medical condition, smoking status, and self-rated health*.

## Discussion

Studies investigating associations of mental health attributes with dietary inflammatory potential using the E-DII scores among university students are rare and those focusing on students residing in university dormitories are not available. In this study, stress and anxiety were significantly associated with the E-DII scores in the female UAEU dormitory residents.

There is an increased interest in the association of dietary inflammatory potential and mental health in adults (35). To the best of our knowledge, this is the first study to focus on university dormitory students aimed at examining the association between diet-associated inflammation and mental health measures including depression, anxiety, and stress.

Relatively high levels of diet-associated inflammation were evident in the dormitory-residing students in this study; the average E-DII score was 2.98 ± 1.17 and values were generally skewed to higher values, ranging from −1.96–5.60, i.e., much higher than typically reported. The recent UAE study on university students mostly living off-campus reported a relatively lower range of the E-DII (36). A comparatively higher E-DII range of −0.09 and 6.1 was reported in female college studying in Saudi Arabia (9), similar values to those found in this study. This difference may be attributed to poor decision-making in food choices among newly independent students residing in dormitories, leaning toward the consumption of less healthy foods compared to students living with families (37).

As expected, the diets of female students in the highest tertile for the E-DII in comparison with the lower tertiles were significantly higher in certain proinflammatory nutrient and dietary components and lower in specific anti-inflammatory food parameters. Previous studies from the UAE and United States also confirmed an increased intakes of proinflammatory dietary factors among university/college students with the higher E-DII scores (36, 38). This should be recognized as an important health barrier and an area for intervention, aimed at improving the diet quality of dormitory students and systems level interventions aimed at incorporating healthier options with anti-inflammatory properties to avoid chronic inflammation and risk of related health problems.

Symptoms of depression (51.9%), anxiety (62.3%), and stress (59.6%) were frequently reported in this study. Comparable incidence rates of mental health disorders were documented among university students in Malaysia (5) and are relatively higher than those reported in another UAE study (9). In contrast, the data contained in the 21 countries used in the WHO World Mental Health Survey Initiative reported much lower rates of mental health disorders (20.3%) in college students aged 18–22 years (2). Higher rates of mental health outcomes in this study warrant institutional actions for the psychological wellbeing of students, especially those residing in university dormitories.

Mental health symptoms for stress and anxiety were significantly higher in students in the highest tertile as compared to tertile 1 of the E-DII. The odds associated with being in tertile 3 for the E-DII for those with symptoms of stress and anxiety were higher (2.88 times and 2.89 times, respectively) than the odds for those in the E-DII tertile 1 for students with no presence of symptoms for stress and anxiety, respectively. Previous studies that measured the associations between stress and/or anxiety and the DII scores among participants supported the current findings (8, 39, 40). To elaborate, Phillips et al. (39) concluded that Irish females with the highest E-DII scores were at elevated risk of showing anxiety symptoms (OR = 2.00, 95% CI: 1.30–3.06, *p* = 0.002) (39). Salari-Moghaddam et al. (40) also showed that Iranian adults in the top quintile of the DII score had greater odds of anxiety (OR = 1.69, 95% CI: 1.07–2.67, *p* = 0.006) and psychological distress (OR = 1.72, 95% CI: 1.20–2.46, *p* = 0.001) than those in the bottom quintile (40). Similarly, the E-DII scores were positively correlated with stress (*r* = 0.46, *p* < 0.0001) in female college students in Saudi Arabia (9).

It is important to put these results in the larger context of the DII–mental health research. There are now over 55 articles and 5 meta-analyses on the DII and depression, 20 articles on the DII and depression, 51 articles on the DII and stress, and 12 articles and one meta-analysis on severe mental illness. Overall, the associations are consistently positive for depression and severe mental illness. They are not as consistent for anxiety (23). So, the results from this study are somewhat inconsistent with previous results. Unlike studies from other regions of the world, no significant association was found in this study between depression and the E-DII scores (39, 41–44). Moreover, recent meta-analysis on depression and the DII scores suggests that proinflammatory diets, estimated by the higher DII score, were independently associated with an increased risk of depression, particularly in females (45). However, the non-significant trend in this study may be attributed to the small sample size, cross-sectional nature of the study, age- and gender-restricted sample, similar environmental conditions in dormitories (campus-life facilities and services), common food catering source for most part of the academic year, and heterogeneity in measurement of depression across different studies. This study employed self-reported assessment of depression, anxiety, and stress in last 1 week that was not a confirmed diagnosis, as these participants were not clinically diagnosed; the results of which may vary with culture among other factors (46). Additionally, it is unknown how accurate these self-reports would be relative to assessment by medical and psychological experts' assessment of depression, anxiety, and stress. Well-designed studies in the future must be designed to help determine whether the association between inflammatory diet and risk of depression in university students is causal. These also should be designed to consider the strong association between repeated episodes of anxiety and chronic long-term anxiety that can lead to depression (47).

Overall, monitoring of mental health of dormitory-residing university students should be considered as an important health policy in educational institutions. Given the strong positive association that has emerged from the DII/E-DII-related research, dietary inflammatory potential should be routinely assessed using validated dietary assessment tools such as 24-h recall interviews and food frequency questionnaires from which the DII scores can be calculated. This could aid in the early detection of the risks of chronic mental health conditions. Institutional administration/management plays a vital role in developing comprehensive systems that may involve targeted intervention strategies combining nutritional-psychological counseling and providing healthy food catering through students' welfare services for promoting health and wellbeing of its residential students, in particular, and all the students, in general.

This study had certain limitations. Despite significant associations evident between dietary inflammatory potential and mental health symptoms, the cross-sectional research design limited causal inference, as it could not rule out reverse causality. Because of the cross-sectional design of this study, it is also unclear whether associations observed reflect short- or longer-term exposure to lower-quality, proinflammatory diets. Likewise, it is not clear whether self-reported experiences of mental health issues in the previous week reflect a long-term characteristic of the participants. Due to time constraints, academic performance could not be included in analyses to determine its potential role in modifying the association between the E-DII scores and mental health of students. Selection of dormitory-residing female students from a single university and lack of comparison with off-campus-residing student counterparts limit the generalization of results. The original concept of the DII scores is based on inclusion of 45 food parameters. However, eight food parameters were excluded due to the lack of food composition values for flavones and isoflavones in local foods and cultural prohibition of alcohol.

Despite some weaknesses, this study has several strengths. The UAEU is the largest national university in the UAE. It is expected that these results will mimic the gender-specific dormitory samples of other universities in the country. The accuracy of the DII scores depends on the dietary assessment used. The administration of 24-h recall method is considered a strong assessment technique. In this study, the 2 days repeated 24-h dietary recalls were taken to reduce the intrapersonal errors, enabling assessment of typical dietary intakes of the participants. Typically, 2 days of recalls produce variance estimates that are smaller than those in structured questionnaires used to measure long-term, habitual intake (48, 49). The DII scores were calculated based on 37 food parameters. In fact, evidence on the DII scores shows strongly consistent findings with the inclusion of as few as 18 food parameters (21). In the future, large-scale prospective research with robust study designs should examine the long-term effects of dietary inflammatory potential, using the DII/E-DII scores on the mental health of university students. This could aid in the early detection of mental disorders and development of nutrition-psychology-based targeted interventions for amelioration of symptoms.

## Conclusion

This study demonstrated that mental health measures, stress and anxiety, were associated with the higher intakes of proinflammatory diets in female students residing in dormitories of the national university in the UAE. Developing targeted interventions such as comprehensive wellness programs on university campus to provide integrated nutritional-psychological support could go a long way in improving the health status of on-campus university students through modulation of dietary options, favoring higher consumption of nutrients and food components with anti-inflammatory effects.

## Data Availability Statement

The dataset supporting the findings of the reported results is available from the corresponding author on reasonable request (AA).

## Ethics Statement

The study was conducted according to the guidelines of the Declaration of Helsinki and approved by the United Arab Emirates Human Research Ethics Committee (Protocol # ERH-2019-5870-19-04).

## Author Contributions

AA: conceptualization, methodology, supervision, writing-original draft, and writing-reviewing and editing. CS: methodology, formal analysis, and writing-reviewing and editing. NS: methodology and software. MW: software and writing-reviewing and editing. MA: investigation and data curation. RA: investigation and data curation. MB: formal analysis and writing-reviewing and editing. JH: methodology, software, and writing-reviewing and editing. All authors contributed to the article and approved the submitted version of the manuscript.

## Conflict of Interest

JH owns controlling interest in Connecting Health Innovations LLC (CHI), a company that has licensed the right to his invention of the DII#x000AE; from the University of South Carolina in order to develop computer and smartphone applications for patient counseling and dietary intervention in clinical settings. NS and MW are employees of the CHI. The subject matter of this article will not have any direct bearing on that work nor has that activity exerted any influence on this project. The remaining authors declare that the research was conducted in the absence of any commercial or financial relationships that could be construed as a potential conflict of interest.

## Publisher's Note

All claims expressed in this article are solely those of the authors and do not necessarily represent those of their affiliated organizations, or those of the publisher, the editors and the reviewers. Any product that may be evaluated in this article, or claim that may be made by its manufacturer, is not guaranteed or endorsed by the publisher.
